# Phase transition on the Si(001) clean surface prepared in UHV MBE chamber: a study by high-resolution STM and *in situ *RHEED

**DOI:** 10.1186/1556-276X-6-218

**Published:** 2011-03-14

**Authors:** Larisa V Arapkina, Vladimir A Yuryev, Kirill V Chizh, Vladimir M Shevlyuga, Mikhail S Storojevyh, Lyudmila A Krylova

**Affiliations:** 1 A. M. Prokhorov General Physics Institute of RAS, 38 Vavilov Street, Moscow, 119991, Russia

**Keywords:** Silicon, Surface reconstruction, Scanning tunnelling microscopy, Reflected high energy electron diffraction, Clean surface preparation

## Abstract

The Si(001) surface deoxidized by short annealing at *T *~ 925°C in the ultrahigh vacuum molecuar beam epitaxy chamber has been in situ investigated using high-resolution scanning tunneling microscopy (STM)and redegreesected high-energy electron diffraction (RHEED. RHEED patterns corresponding to (2 × 1) and (4 × 4) structures were observed during sample treatment. The (4 × 4) reconstruction arose at *T *≲ 600°C after annealing. The reconstruction was observed to be reversible: the (4 × 4) structure turned into the (2 × 1) one at *T *≳ 600°C, the (4 × 4)  structure appeared again at recurring cooling. The *c*(8 × 8) reconstruction was revealed by STM at room temperature on the same samples. A fraction of the surface area covered by the *c*(8 × 8) structure decreased, as the sample cooling rate was reduced. The (2 × 1) structure was observed on the surface free of the *c*(8 × 8) one. The *c*(8 × 8) structure has been evidenced to manifest itself as the (4 × 4) one in the RHEED patterns. A model of the *c*(8 × 8) structure formation has been built on the basis of the STM data. Origin of the high-order structure on the Si(001) surface and its connection with the epinucleation phenomenon are discussed.

**PACS **68.35.B-·68.37.Ef·68.49.Jk·68.47.Fg

## Introduction

Investigations of clean silicon surfaces prepared in conditions of actual technological chambers are of great interest due to the industrial requirements to operate on nanometer and subnanometer scales when designing future nanoelectronic devices [1]. In the nearest future, the sizes of structural elements of such devices will be close to the dimensions of structure features of Si(001) surface, at least of its high-order reconstructions such as *c*(8 × 8). Most of researches of the Si(001) surface have thus far been carried out in specially refined conditions which allowed one to study the most common types of the surface reconstructions such as (2 × 1), *c*(4 × 4), *c*(4 × 2), or *c*(8 × 8) [2-14]. Unfortunately, no or a very few papers have thus far been devoted to investigations of the Si surface which is formed as a result of the wafer cleaning and deoxidation directly in the device manufacturing equipment [14]. However, anyone who deals with Si-based nanostructure engineering and the development of such nanostructure formation cycles compatible with some standard device manufacturing processes meets the challenging problem of obtaining the clean Si surface within the imposed technological restrictions which is one of the key elements of the entire structure formation cycle [1,15,16].

The case is that the ambient in technological vessels such as molecular beam epitaxy (MBE) chambers is usually not as pure as in specially refined ones designed for surface studies. There are many sources of surface contaminants in the process chambers including materials of wafer heaters or evaporators of elements as well as foreign substances used for epitaxy and doping. In addition, owing to technological reasons, the temperature treatments applicable for device fabrication following the standard processes such as CMOS often cannot be as aggressive as those used for surface preparation in the basic experiments. Moreover, the commercially available technological equipment sometimes does not realize the wishful annealing of Si wafers at the temperature of ~1200°C even if the early device-formation stage allows one to heat the wafer to such a high temperature. Nevertheless, the technologist should always be convinced that the entirely deoxidized and atomically clean Si surface is reliably and reproducibly obtained.

A detailed knowledge of the Si surface structure which is formed in the above conditions--its reconstruction, defectiveness, fine structural peculiarities, etc.--is of great importance too, because this structure may affect the properties of nanostructured layers deposited on it. For instance, the Si surface structure may affect the magnitude and the distribution of the surface stress of the Ge wetting layer on nanometer scale when the Ge/Si structure is grown, which in turn affects the Ge nanocluster nucleation and eventually the properties of quantum dot arrays formed on the surface [1,16-30].

Thus, it is evident from the above that the controllable formation of the clean Si(001) surface with the prescribed parameters required for technological cycles of nanofabrication compatible with the standard device manufacturing processes should be considered as an important goal, and this article paves the way for the same.

In this article, we report the results of investigation of the Si(001) surface treated following the standard procedure of Si wafer preparation for the MBE growth of the SiGe/Si(001) or Ge/Si(001) heterostructures. A structure arising on the Si(001) surface as a result of short high-temperature annealing for SiO_2 _removal is explored. It is well known that such experimental treatments favor the formation of nonequilibrium structures on the surface. The most studied of them are presently the (2 × 1) and *c*(4 × 4) structures. This study experimentally investigates by means of scanning tunneling microscopy (STM) and reflected high-energy electron diffraction (RHEED) the formation and atomic structure of the less-studied high-order *c*(8 × 8) or *c*(8 × *n*) [14-16]) reconstruction. Observations of this reconstruction have already been reported in the literature [4-6,10], but there is no clear comprehension of causes of its formation as the structures looking like the *c*(8 × 8) one appear after different treatments: The *c*(8 × 8) reconstruction was observed to be a result of the copper atoms deposition on the Si(001)-(2 × 1) surface [7,10]; similar structures were found to arise because of various treatments and low-temperature annealing of the original Si(001)-(2 × 1) surface without deposition of any foreign atoms [4-6]. Data of the STM studies of the Si(001)-*c*(8 × 8) surface were presented in refs. [5,10].

It may be supposed on the analogy with the Si(001)-*c*(4 × 4) reconstruction [12,31-35] that the presence of impurity atoms on the surface as well as in the subsurface regions is not the only reason for the of formation of reconstructions different from the (2 × 1) one, but the conditions of thermal treatments also should be taken into account. The results of exploration of effect of such factor as the rate of sample cooling from the annealing temperature to the room temperature on the process of the *c*(8 × 8) reconstruction formation are reported in this article. It is shown by means of RHEED that the diffraction patterns corresponding to the (2 × 1) surface structure reversibly turn into those corresponding to the *c*(8 × 8) one depending on the sample temperature, and a point of this phase transition is determined. Based on the STM data, a model of the *c*(8 × 8) structure formation is brought forward.

## Methods and equipment

The experiments were conducted using an integrated ultra-high-vacuum (UHV) system [27] based on the Riber EVA 32 MBE chamber equipped with the Staib Instruments RH20 diffractometer of reflected high-energy electrons and coupled through a transfer line with the GPI 300 UHV scanning tunnelling microscope [36-38]. This instrument enables the STM study of samples at any stage of Si surface preparation and MBE growth. The samples can be serially moved into the STM chamber for the analysis and back into the MBE vessel for further treatments as many times as required never leaving the UHV ambient. RHEED experiments can be carried out *in situ*, i.e., directly in the MBE chamber during the process.

Samples for STM were 8 × 8 mm^2 ^squares cut from the specially treated commercial B-doped CZ Si(100) wafers (*p*-type, *ρ *= 12 Ω cm). RHEED measurements were carried out on the STM samples and similar 2*" *wafers; the 2*" *samples were investigated only by means of RHEED. After chemical treatment following the standard procedure described elsewhere [1,39] (which included washing in ethanol, etching in the mixture of HNO_3 _and HF and rinsing in the deionized water), the samples were placed in the holders. The STM samples were mounted on the molybdenum STM holders and inflexibly clamped with the tantalum fasteners. The STM holders were placed in the holders for MBE made of molybdenum with tantalum inserts. The 2*" *wafers were inserted directly into the standard molybdenum MBE holders and did not have so much hard fastening as the STM samples.

**Figure 1:**
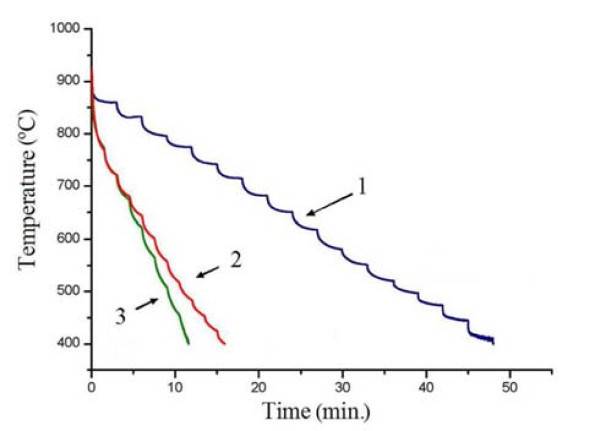
**A diagram of sample cooling after the thermal treatment at 925°C measured by IR pyrometer; cooling rates are as follows**: ~0.17°C/s or "slow cooling" of the STM samples (1); ~0.4°C/s or "quenching" of the STM samples (2) and 2*" *wafers (3).

Afterward, the samples were loaded into the airlock and transferred into the preliminary annealing chamber where outgassed at ~600°C and ~5 × 10^-9 ^Torr for about 6 h. Then, the samples were moved for final treatment, and decomposition of the oxide film into the MBE chamber evacuated down to ~10^-11 ^Torr. There were two stages of annealing in the process of sample heating: at ~600°C for ~5 min and at ~800°C for ~3 min [1,14,27]. The final annealing was carried out at ~925°C.^1 ^Then, the temperature was rapidly lowered to ~850°C. The rates of the further cooling down to the room temperature were ~0.4°C/s (referred to as the "quenching" mode of both the STM samples and 2*" *wafers) or ~0.17°C/s (called the "slow cooling" mode of only the STM samples) (Figure 1). The pressure in the MBE chamber increased to ~2 × 10^-9 ^Torr during the process.

In both chambers, the samples were heated from the rear side by radiators of tantalum. The temperature was monitored using the IMPAC IS 12-Si pyrometer which measured the Si sample temperature through chamber windows. The atmospheric composition in the MBE chamber was monitored using the SRS RGA-200 residual gas analyzer before and during the process.

After cooling, the STM samples were moved into the STM chamber in which the pressure did not exceed 1 × 10^-10 ^Torr. RHEED patterns were obtained for all the samples directly in the MBE chamber at different elevated temperatures during the sample thermal treatment and at room temperature after cooling. The STM samples were always explored by RHEED before moving into the STM chamber.

The STM tips were *ex situ *made of the tungsten wire and cleaned by ion bombardment [40] in a special UHV chamber connected to the STM chamber. The STM images were obtained in the constant tunnelling current mode at room temperature. The STM tip was zero-biased, while the sample was positively or negatively biased when scanned in empty- or filled-states imaging mode.

The STM images were processed afterward using the WSxM software [41].

## Experimental findings

**Figure 2:**
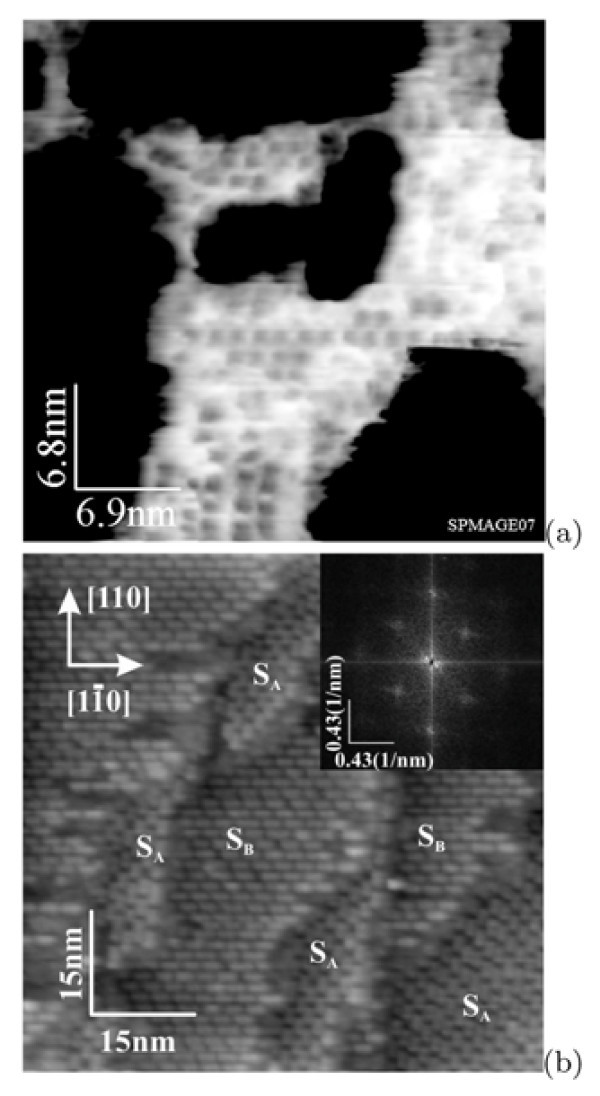
**STM images of the Si(001) surfaces**: **(a) **the surface with the residual silicon oxide (-1.5 V, 150 pA), annealing at ~925°C for ~2 min; the image is inverted: dark areas correspond to the oxide, the lighter areas represent the deoxidized surface; **(b) **the clean Si(001) surface (+1.9 V, 70 pA) with the Fourier transform pattern shown in the inset, annealing at ~925°C for ~3 min [14].

Figure 2 demonstrates the STM images of the Si(001) surface after annealing at ~ 925°C of different durations. Figure 2a depicts the early phase of the oxide film removal; the annealing duration is 2 min. A part of the surface is still oxidized: the dark areas in the image correspond to the surface coated with the oxide film. The lighter areas correspond to the purified surface. A structure of ordered "rectangles" (the grey features) is observed on the deoxidizes surface. After longer annealing (for 3 min) and quenching (Figure 1), the surface is entirely purified of the oxide (Figure 2b). It consists of terraces separated by the *S*_B _or *S*_A _monoatomic steps with the height of ~1.4 Å [3]. Each terrace is composed of rows running along [110] or [] directions. The surface reconstruction is different from the (2 × 1) one. The inset of Figure 2b demonstrates the Fourier transform of this image which corresponds to the *c*(8 × 8) structure [5]: Reflexes of the Fourier transform correspond to the distance ~31 Å in both [110] and [] directions. Therefore, the revealed structure have a periodicity of ~31 Å that corresponds to eight translations *a *on the surface lattice of Si(001) along the 〈110〉 directions (*a *= 3.83 Å is a unit translation length). Rows consisting of structurally arranged rectangular blocks are clearly seen in the empty-state STM image (Figure 2b). They turn by 90° on the neighboring terraces.

**Figure 3:**
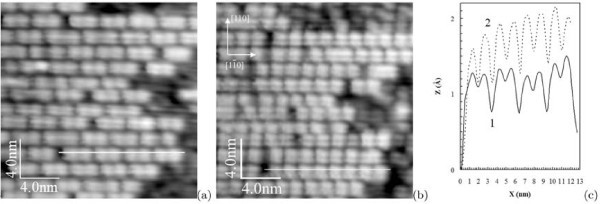
**STM images and line scans of the same region on the Si(001) surface**: **(a) **empty states (+1.7 V, 100 pA) and **(b) **filled states (-2.0 V, 100 pA); positions of extremes of line scan profiles **(c) **match exactly for the empty- (1) and filled-state (2) distributions along the corresponding lines in the images **(a) **and **(b)**.

Figure 3 demonstrates the empty- and filled-state images of the same surface region. Each block consists of two lines separated by a gap. This fine structure of blocks is clearly seen in the both pictures (a) and (b), but its images are different in different scanning modes. A characteristic property most clearly seen in the filled-state mode (Figure 3b) is the presence of the brightness maxima on both sides of the lines inside the blocks. These peculiar features are described later in more detail. Figure 3c shows the profiles of the images taken along the white lines. Extreme positions of both curves are well fitted. Relative heights of the features outside and inside the blocks can be estimated from the profiles.

**Figure 4:**
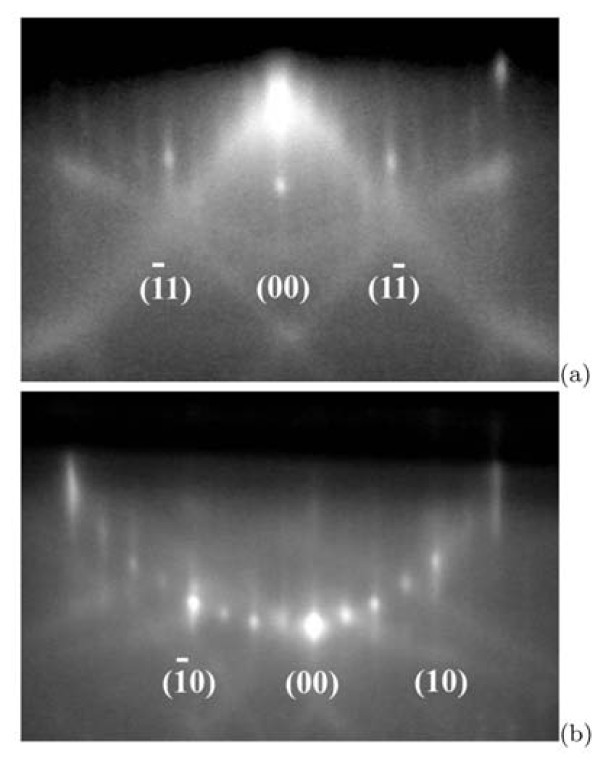
**Reflected high-energy electron diffraction patterns**: **(a) **[0 1 0] and **(b) **[1 1 0] azimuths; electron energies were 9.8 and 9.3 keV, respectively.

Figure 4 demonstrates typical RHEED patterns taken at room temperature from the STM sample annealed for 3 min with further quenching. Characteristic distances on the surface corresponding to the reflex positions in the diffraction pattern were calculated according to ref. [42]. The derived surface structure is (4 × 4). One sample showed the RHEED patterns corresponding to the (2 × 1) structure [42] after the same treatment however.

Temperature dependences of the RHEED patterns in the [110] azimuth were investigated during sample heating and cooling. It was found that the reflexes corresponding to 2*a *were distinctly seen in the RHEED patterns during annealing at ~925°C after 2 min of treatment. The reflexes corresponding to 4*a *started to appear during sample quenching and became definitely visible at the temperature of ~600°C; a weak (4 × 4) signal started to arise at ~525°C if the sample was cooled slowly (Figure 1). At the repeated heating from room temperature to 925°C, the (4 × 4) structure disappeared at ~600°C giving place to the (2 × 1) one. The (4 × 4) structure appeared again at ~600° during recurring cooling.

The RHEED patterns obtained from 2*" *samples always corresponded to the (2 × 1) reconstruction. Diffraction patterns for the STM sample which was not hard fastened to the holder corresponded to the (4 × 4) structure after quenching (STM measurements were not made for this sample).

**Figure 5:**
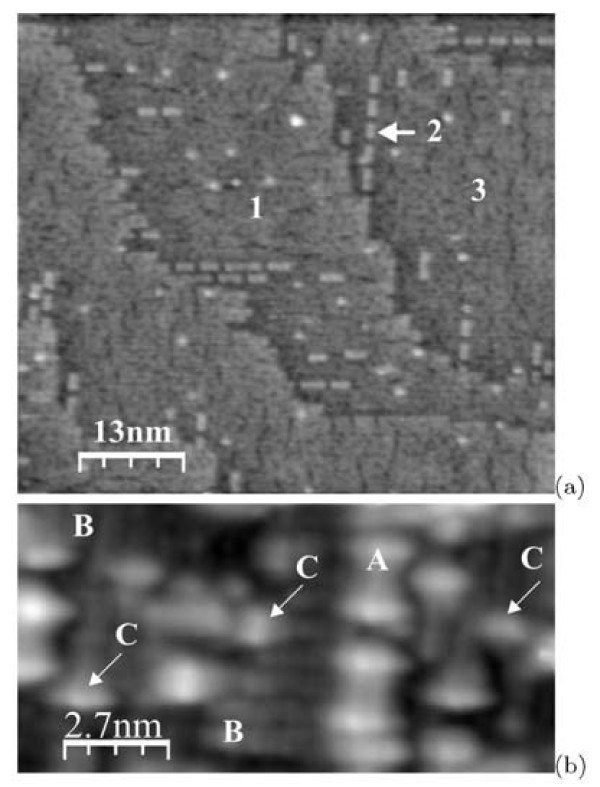
**STM images of the clean Si(001) surface prepared in the slow-cooling mode**: **(a) **the surface mainly covered by the (2 × 1) structure (+2.0 V, 100 pA), '1' and '3' are terraces; the height of the row '2' coincides with the height of the terrace '1'; **(b) **a magnified image taken with atomic resolution (-1.5 V, 150 pA), 'A' is the "rectangle", 'B' marks the dimer rows composing the (2 × 1) structure (*separate atoms are seen*), 'C' shows structural defects, i.e. the dimers of the uppermost layer oriented along the dimers of the lower (2 × 1) rows.

Effects of annealing duration and cooling rate on the clean surface structure were studied using STM. It was established that increase of annealing duration to 6 min did not cause any changes of the surface structure. On the contrary, decrease of the sample cooling rate drastically changes the structure of the surface. The STM images of the sample surface for the slow-cooling mode (Figure 1) are presented in Figure 5. The difference of this surface from that of the quenched samples (Figure 2b) is that only a few rows of "rectangles" are observed on it. The order of the "rectangle" positions with the period of 8*a *remains in such rows. Two adjacent terraces are designated in Figure 5a by '1' and '3'. A row of "rectangles" marked as '2' is situated on the terrace '3'; it has the same height as the terrace '1'. The filled-state image, which is magnified in comparison with the former one, is given in Figure 5b. A part of the surface free of the "rectangles" is occupied by the (2 × 1) reconstruction. Images of the dimer rows with the resolved Si atoms are marked as 'B' in Figure 5b. The "rectangles" are also seen in the image (they are marked as 'A') as well as single defects: dimerized Si atoms ('C') and chaotically located on the surface accumulations of several dimers. Most of these dimers are oriented parallel to dimers of the lower surface and located strictly on the dimer row. The influence of the cooling rate on the surface structure was observed by Kubo et al. [6]: when the sample cooling rate was decreased, the surface reconstruction turned from *c*(8×8) to *c*(4 × 2), which was considered as the derivative reconstruction of the (2 × 1) one transformed because of dimer buckling.

**Figure 6:**
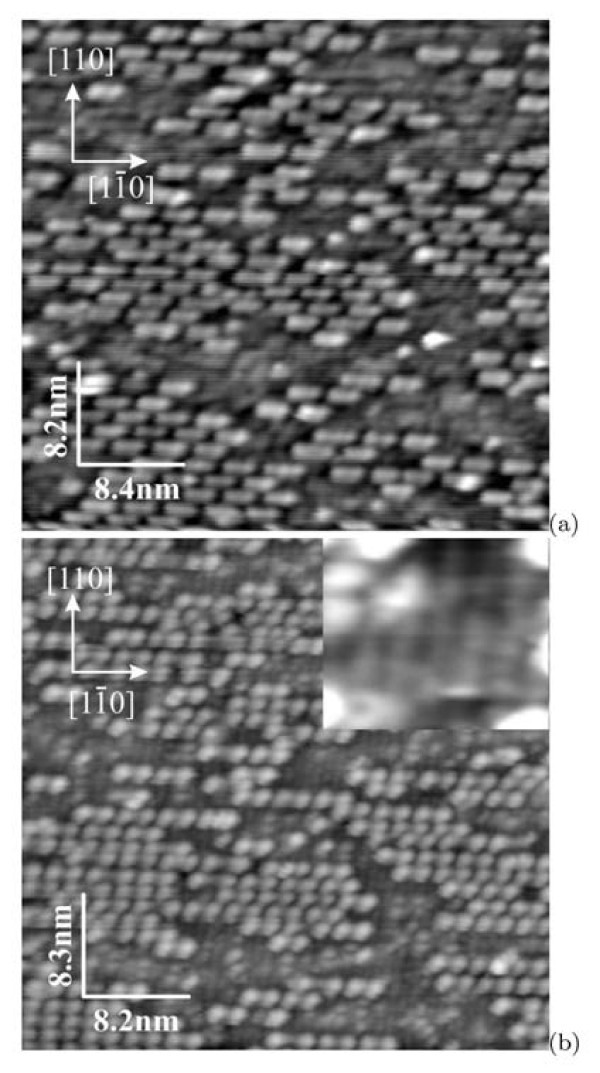
**STM images of the same region on the Si(001) surface**: **(a) **empty states (+2.0 V, 100 pA) and **(b) **filled states (-2.0 V, 100 pA); an inset at **(b) **shows the image of the (2 × 1) surface between the rows of "rectangles".

Figure 6 presents the STM images obtained for the samples cooled in the quenching mode but containing areas free of "rectangles". The images (a) and (b) of the same place on the surface were obtained serially one by one. We managed to image the surface structure between the areas occupied by the "rectangle" rows, but only in the filled-state mode (see the inset at Figure 6b). Similar to as shown in Figure 5b, this structure is seen to be formed by parallel dimer rows going 2*a *apart. The direction of these rows is perpendicular to the direction of the rows of "rectangles". The height difference of the rows of "rectangles" and the (2 × 1) rows is 1 monoatomic step (~1.4Å). We did not succeed to obtain a good enough image of these subjacent dimer rows in the empty-state mode. It should be noted also that positions of the "rectangles" are always strictly fixed relative to the dimer rows of the lower layer: they occupy exactly three subjacent dimer rows. It also may be seen in the STM images presented in refs. [5,10].

### Fine structure of the observed reconstruction

Let us consider the observed structure in detail.

A purified sample surface consists of monoatomic steps. Following the nomenclature by Chadi [3], they are designated as *S*_A _and *S*_B _in Figure 2b. Each terrace is composed of rows running along the [110] or [] directions. Each row consists of rectangular blocks ("rectangles"). They may be regarded as surface structural units, as they are present on the surface after thermal treatment in any mode, irrespective of a degree of surface coverage by them. Reflexes of the Fourier transform of the picture shown in Figure 2b correspond to the distances ~31 and ~15 Å in both [110] and [] directions. Hence, the structure revealed in the long shot seems to have a periodicity of ~31 Å, which corresponds to eight translations *a *on the surface lattice of Si(001). It resembles the Si(001)-*c*(8 × 8) surface [5]. Reflexes corresponding to the distance of ~15 Å (4*a*) arise because of the periodicity along the rows. STM images obtained at higher magnifications give an evidence that the surface appears to be disordered, however.

**Figure 7:**
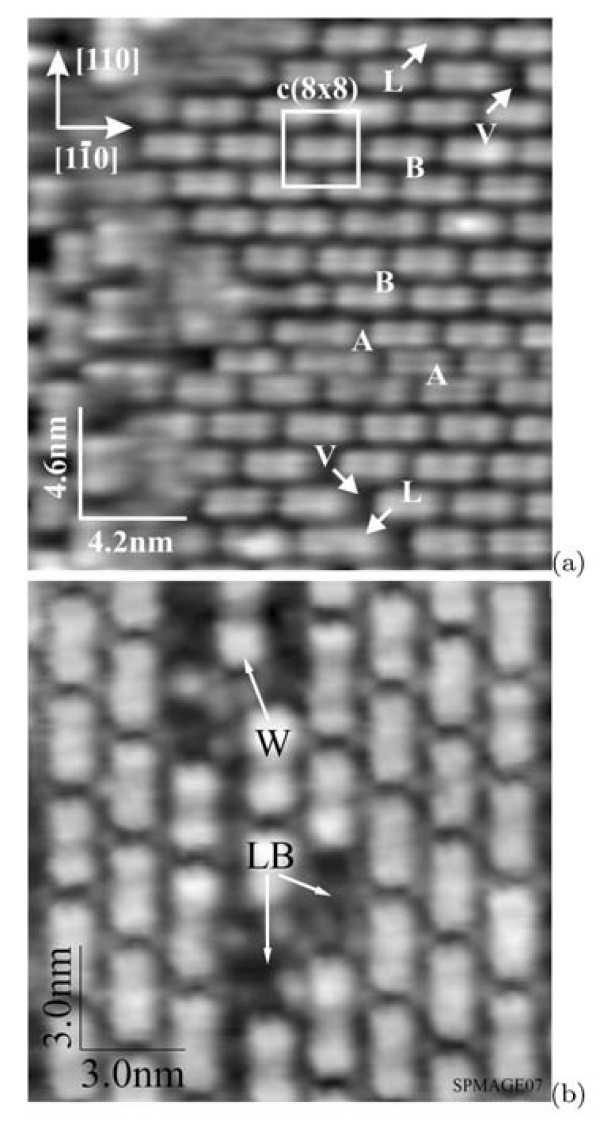
**STM empty-state images of the Si(001) surface**: a *c*(8 × 8) unit cell is marked by the white box in image **(a) **(+1.9 V, 50 pA), distances between the rows marked by 'A' and 'B' equal 3*a *and 4*a *(which correspond to *c*(8 × 6) and *c*(8 × 8) structures, respectively), two long "rectangles" and divacancies arising in the adjacent rows are marked by 'L' and 'V', respectively; a row wedging between two rows ('W') and lost blocks ('LB') are seen in **(b) **(+1.6 V, 100 pA).

Figure 7 shows the magnified images of the investigated surface. The rows of the blocks are seen to be situated at varying distances from one another (hereinafter, the distances are measured between corresponding maxima of features). A unit *c*(8 × 8) cell is marked with a square box in Figure 7a. The distances between the adjacent rows of the rectangles are 4*a *in such structures ('B' in Figure 7a). The adjacent rows designated as 'A' are 3*a *apart (*c*(8 × 6)).

A structure with the rows going at 4*a *apart is presented in Figure 7b. The lost blocks ('LB') that resemble point defects are observed in this image. In addition, a row wedging in between two rows and separating them by an additional distance *a *is seen in the center of the upper side of the picture ('W'). The total distance between the wedged off rows becomes 5*a*.

Hence it may be concluded that the order and some periodicity take place only along the rows--disordering of the *c*(8 × 8) structure across the rows is revealed (we often refer to this structure as *c*(8 × *n*)).

The block length can possess two values: ~15 Å (4*a*) and ~23 Å (6*a*). Distances between equivalent positions of the adjacent short blocks in the rows are 8*a*. If the long block appears in a row, a divacancy is formed in the adjacent row to restore the checkerboard order of blocks. Figure 7a illustrates this peculiarity. The long block is marked as 'L', the divacancy arisen in the adjacent row is lettered by 'V'. In addition, the long blocks were found to have one more peculiarity. They have extra maxima in their central regions. The maxima are not so pronounced as the main ones but nevertheless they are quite recognizable in the pictures (Figure 7a).

**Figure 8:**
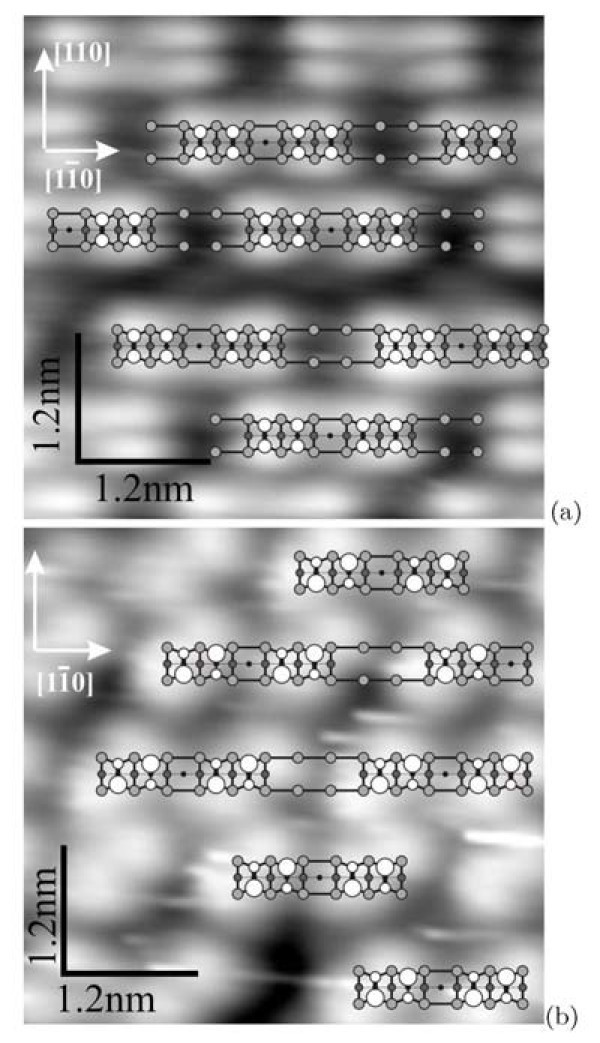
**STM images of the Si(001)-*c*(8 × *n*) surface**: **(a) **empty states (+1.7 V, 150 pA) and **(b) **filled states (-2.2 V, 120 pA). Corresponding schematic drawings of the surface structure are superimposed on both pictures. The lighter circle the higher the corresponding atom is situated in the surface structure. The dimer buckling is observed in the filled state image **(b)**, which is reflected in the drawing by larger open circles representing higher atoms of the tilted Si dimers of the uppermost layer of the structure.

Figure 8 presents magnified STM images of the blocks ("short rectangles"). The images obtained in the empty-state (Figure 8a) and filled-state (Figure 8b) modes are different. In the empty-state mode, short blocks look like two lines separated by ~8 Å (the distance is measured between brightness maxima in each line). It is the maximum measured value which can lessen depending on scanning parameters. Along the rows, each block is formed by two parts. The distance between the brightness maxima of these parts is ~11.5 Å (or some greater depending on scanning parameters). In the filled-state mode, the block division into two structurally identical parts remains. Depending on scanning conditions, each part looks like either coupled bright dashes and blobs (Figure 3b, 6b) or two links (brightness maxima) of zigzag chains (Figure 8b). The distances between the maxima are ~4 Å along the rows.

The presented STM data are interpreted by us to correspond with a structure composed of Si ad-dimers and divacancies.

## Discussion

### Structural model

The above data allow us to bring forward a model of the observed Si(001) surface reconstruction. The model is based on the following assumptions: (i) the outermost surface layer is formed by ad-dimers; (ii) the underlying layer has a structure of (2×1); and (iii) every rectangular block consists of ad-dimers and divacancies a number of which control the block length.

**Figure 9:**
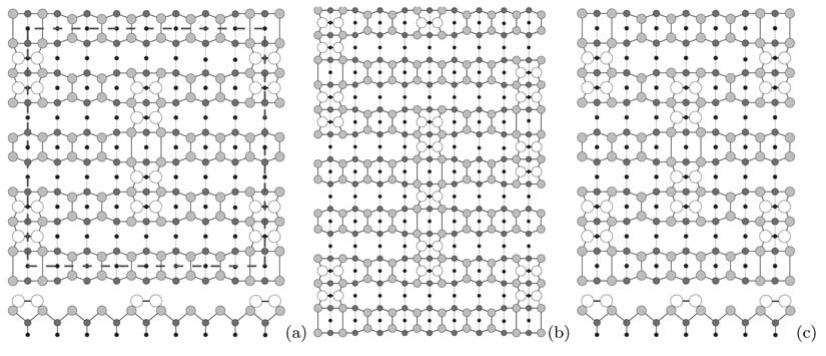
**A schematic drawing of the *c*(8 × *n*) structure**: **(a) ***c*(8 × 8) with the short blocks, a unit cell is outlined; **(b) **the same structure with the long block; **(c) ***c*(8 × 6) structure.

Figure 9a shows a schematic drawing of the *c*(8 × 8) structure (a unit cell is outlined). This structure is a basic one for the model brought forward. The elementary structural unit is a short rectangle. These blocks form raised rows running vertically (shown by empty circles). Smaller shaded circles show horizontal dimer rows of the lower terrace. The remaining black circles show bulk atoms. Each "rectangle" consists of two dimer pairs separated with a dimer vacancy. The structures on the Si(001) surface composed of close ad-dimers are believed to be stable [6,13] or at least metastable [43]. In our model, a position of the "rectangles" is governed by the location of the dimer rows of the (2 × 1) structure of the underlying layer. The rows of blocks are always normal to the dimer rows in the underlying layer to form a correct epiorientation [43]. Every rectangular block is bounded by the dimer rows of the underlying layer from both short sides. Short sides of blocks form non-rebonded *S*_B _steps [3] with the underlying substrate (see Figure 5b, and three vertically running (the very left) rows of "rectangles" in Figure 7a).

Figure 9b demonstrates the same model for the case of the long rectangle. This block is formed because of the presence of an additional dimer in the middle of the rectangle. The structure consisting of one dimer is metastable [6,13], and so this type of blocks cannot be dominating in the structure. Each long block is bounded on both short sides by the dimer rows of the underlying terrace, too. The presence of the long rectangle results in the formation of a dimer-vacancy defect in the adjacent row; this is shown in Figure 9b--the long block is drawn in the middle row, while the dimer vacancy is present in the last left row. According to our STM data, the surface is disordered in the direction perpendicular to the rows of the blocks. The distances between the neighboring rows may be less than those in the *c*(8 × 8) structure. Hence, the structure presented in this article may be classified as *c*(8×*n*) one. Figure 9c demonstrates an example of such structure--the *c*(8 × 6) one.

In Figure 8 the presented structure is superimposed on STM images of the surface. The filled-state image (Figure 8b) reveals dimer buckling in the blocks which is often observed in this mode at some values of sample bias and tunneling current. Upper atoms of tilted dimers are shown by larger open circles.

### Comparison of STM and RHEED data

**Figure 10:**
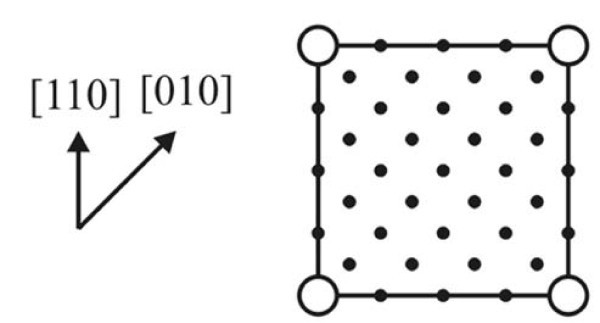
**The Si(001)-*c*(8 × 8) surface reciprocal lattice**.

**Figure 11:**
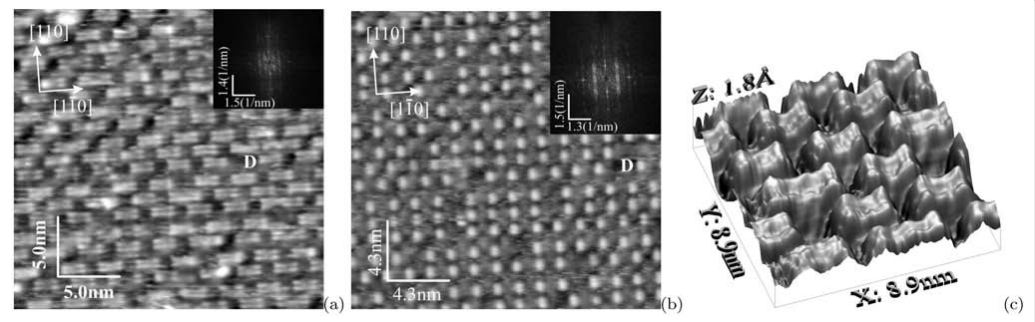
**STM images of the Si(001) surface**: the images of the same area obtained in the **(a) **empty-state (+1.96 V, 120 pA) and **(b) **filled-state (-1.96 V, 100 pA) modes; for the convenience of comparison, 'D' indicates the same vacancy defect; corresponding Fourier transforms are shown in the insets. A 3D STM empty-state micrograph (+2.0 V, 200 pA) of the Si(001)-*c*(8 × 8) surface is shown in **(c)**.

Now we shall explain the observed discrepancy of results obtained by STM and RHEED within the proposed model. Figure 10 presents a sketch of the reciprocal lattice of *c*(8 ×8). The RHEED patterns obtained in the [110] azimuth correspond to the *c*(8 × 8) structure; the patterns observed in the [010] azimuth do not (Figure 4). The reason for this discrepancy may be understood from the filled-state STM image which corresponds to the electron density distribution of electrons paired in covalent bond of a Si-Si dimer. Figure 11 compares STM images of the same region on one terrace obtained in the empty-state (a) and filled-state (b) modes; insets show their Fourier transforms, the differences in which for the two STM modes are as follows: in the Fourier transform of the filled-state image, reflexes corresponding to the distance of 8*a *are absent in the [110] and [] directions, whereas the reflexes corresponding to 4*a *and 2*a *are present (it should be noticed that the image itself resembles that of the (4 × 4) reconstructed surface). If an empty-state image is not available, then it might be concluded that the (4 × 4) structure is arranged on the surface. An explanation of this observation is simple. Main contribution to the STM image is made by ad-dimers situated on the sides of the "rectangles", i.e., on tops of the underlying dimer rows. According to calculations made, e.g., in refs. [44,45], dimers located in such a way are closer to the STM tip and appear in the images to be brighter than those situated in the troughs. Hence, it may be concluded that the RHEED (4 × 4) pattern results from electron diffraction on the extreme dimers of the "rectangles" forming the *c*(8 × 8) surface structure.

The latter statement is illustrated by the STM 3D empty-state topograph shown in Figure 11c. The extreme dimers located on the sides of the rectangular blocks are seen to be somewhat higher than the other ones of the dimer pairs; they form a superfine relief which turned out to be sufficient to backscatter fast electrons incident on the surface at grazing angles.

### Origin

The Si(001)-*c*(8 × 8) structure have formerly been observed and described in a number of publications [4-7,10]. Conditions of its formation were different: copper atoms were deposited on silicon (2 × 1) surface to form the *c*(8 × 8) reconstruction [10], although it is known that Cu atoms are not absorbed on the Si(001) clean surface if the sample temperature is greater than 600°C, and on the contrary Cu desorption from the surface takes place [7,10]; fast cooling from the annealing temperature of ~1100°C was applied [4,5]; samples treated in advance by ion bombardment were annealed and rapidly cooled [6]. The resultant surfaces were mainly explored by STM and low-energy electron diffraction. STM investigations yielded similar results-- a basic unit of the reconstruction was a "rectangle", but the structure of the "rectangles" revealed by different authors was different. In general, an origin of the Si(001)-*c*(8 × 8) structure is unclear until now.

STM images that most resembled our data were reported in ref. [5]. In that article, the *c*(8 × 8) structure was observed in samples without special treatment by copper: the samples were subjected to annealing at the temperature of ~1050°C for the oxide film removal. Formation of the *c*(8 × 8) reconstruction was explained in that article by the presence of a trace amount of Cu atoms the concentration of which was beyond the Auger electron spectroscopy detection threshold. The STM empty-state images of the samples were similar to those presented in this article. A very important difference is observed in the filled-state images--we observe absolutely different configuration of dimers within the "rectangles". Nevertheless, the presence of Cu cannot be completely excluded. Some amount of the Cu atoms may come on the surface from the construction materials of the MBE chamber (although there is a circumstance that to some extent contradicts this viewpoint: Cu atoms were not detected in the residual atmosphere of the MBE chamber within the sensitivity limit of the SRS RGA-200 mass spectrometer) or even from the Si wafer. Cu is known to be a poorly controllable impurity, and its concentration in the subsurface layers of Si wafers which were not subjected to the gettering process may reach 10^15 ^cm^-3^. This amount of Cu may appear to be sufficient to give rise to the formation of the defect surface reconstruction. However, the following arguments urge us to doubt about the Cu-based model: (i) undetectable trace amounts of Cu were suggested in ref. [5], the presence or absence of which is unprovable; (ii) even if the suggestion is true, our STM images give an evidence of a different amount of dimers in the rectangular blocks; so, it is unclear why Cu atoms form different stable configurations on similar surfaces; and (iii) it is hard to explain why Cu atoms cyclically compose and decompose the rectangular blocks during the cyclical thermal treatments of the samples. It applies equally to any other impurity or contamination.

Now, we consider a different interpretation of our data. As mentioned above, the literature suggests two causes of *c*(8 × 8) appearance. The first is an impact of impurity atoms adsorbed on the surface even at trace concentrations. The second is a thermal cycle of the oxide film decomposition and sample cooling. The first model seems to be hardly applicable for explanation of the reported experimental results. According to our data, no impurities are adsorbed directly on the studied surface: RHEED patterns correspond to a clean Si(001) surface reconstructed in (2 × 1) or, at lower temperatures, (4 × 4) configuration. Cyclic contaminant desorption at high temperatures (≳ 600°C) and adsorption on sample cooling is unbelievable. Consecutive segregation and desegregation of an undetectable impurity in sub-surface layers also does not seem verisimilar.

The second explanation looks more attractive. It was found in ref. [46] as a result of the STM studies that the Si(001) surface subjected to the thermal treatment at ~820°C which was used for decomposition of the thin (~1 nm thick) SiO_2 _films obtained by chemical oxidation contained a high density of vacancy-type defects and their agglomerates as well as individual ad-dimers. Therefore, the initial bricks for the considered surface structure are abundant after the SiO_2 _decay.

The literature presents a wide experimental material on a different reconstruction of the Si(001) surface--*c*(4 × 4)--which also arise at the temperatures of ≳ 600°C. For example, a review of articles describing different experimental investigations can be found in refs. [12,31-35]. Based on the generalized data, an inference can be made that the *c*(4 × 4) structure forms in the interval from 600 to 700°C. Most likely, at these temperatures, an appreciable migration of Si ad-atoms starts on surface. The structure is free of impurities. It irreversibly transits to the (2 × 1) one at the temperature greater than 720°C. Aruga and Murata [47] demonstrate formation of the Si(001)-(2 × 8) structure, also without impurity atoms. In analogy with the above literature data, formation of the *c*(8 × 8) reconstruction may be expected as a result of low-temperature annealing and/or further quenching. The standard annealing temperature for obtaining (2 × 1) structure is known to be in the interval from 1200 to 1250°C. At these temperatures in UHV ambient, not only oxide film removal from the surface takes place, but also silicon evaporation and carbon desorption go on. Unfortunately, we have not got a technical opportunity to carry out such a high-temperature annealing in our instrument. Treatment at 925°C that we apply likely does not result in substantial evaporation of Si atoms from the surface, and C atoms, if any, may diffuse into subsurface layers. As a result, a great amount of ad-dimers arise on the surface, like it happens in the process described in ref. [46]. Formation processes of the (2 × 1) and *c*(8 × 8) structures are different. The (2 × 1) reconstruction arises during the high-temperature annealing, and ad-atoms of the uppermost layer do not need to migrate and be embedded into the lattice to form this reconstruction. On the contrary, *c*(8 × 8) appears during sample cooling, at rather low temperatures, and at the moment of a prior annealing the uppermost layer consists of abundant ad-atoms. On cooling, the ad-dimers have to migrate along the surface and be built in the lattice. A number of competing sinks may exist on the surface (steps, vacancies, etc.), but high cooling rate may impede ad-atom annihilation slowing their migration to sinks and in such way creating supersaturation and favoring 2D islanding, and freezing a high-order reconstruction.

**Figure 12:**
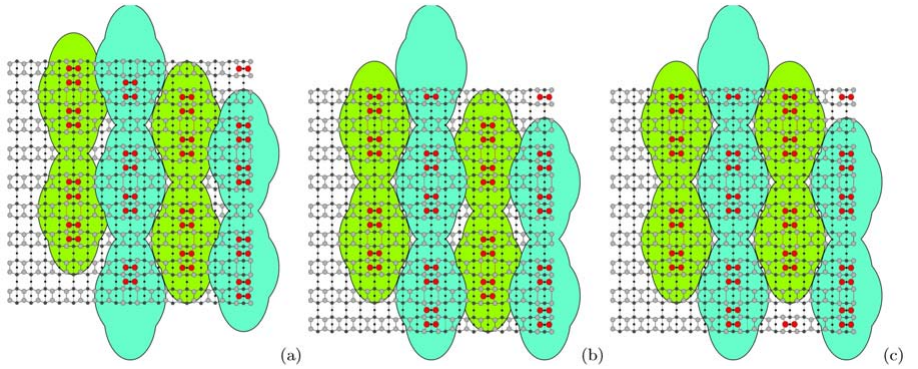
**Schematic representation of the surface stress fields interactions during formation of the *c*(8 × 8) structure**: **(a) **ordering of the "rectangles" within the rows; **(b) **ordering of the rows relative to each other; **(c) **the ordered *c*(8 × 8) structure.

The following scenario may be proposed to describe the *c*(8 × 8) structure formation. A large number of ad-dimers remains on the surface during the sample annealing after the oxide film removal. They form the uppermost layer of the structure. The underlying layer is (2 × 1) reconstructed. Ad-dimers are mobile and can form different complexes (islands). Calculations show that the most energetically favorable island configurations are single dimer on a row in non-epitaxial orientation [43,45,48,49] (Figure 5b), complexes of two dimers (pairs of dimers) in epiorientation (metastable [43]) and two dimers on a row in non-epitaxial orientation separated by a divacancy, and tripple-dimer epi-islands considered as critical epinuclei [43]. These mobile dimers and complexes migrate in the stress field of the (2 × 1) structure. The sinks for ad-dimers are (A) steps, (B) vacancy defects of the underlying (2 × 1) reconstructed layer, and (C) "fastening" them to the (2 × 1) surface as a *c*(8 × 8) structure. The main sinks at high temperatures are A and B. As the sample is cooled, the C sink becomes dominating. Ad-dimers on the Si(001)-(2 × 1) surface are known to tend to form dimer rows [50]. In this case, such rows are formed by metastable dimer pairs gathered in the "rectangles". The "rectangles" are ordered with a period of eight translations in the rows. The ordering is likely controlled by the (2 × 1) structure of the underlying layer and interaction of the stress fields arising around each "rectangle". Effect of the underlying (2 × 1) layer is that the "rectangle" position on the surface relative to its dimer rows is strictly defined: dimers of the "rectangle" edges must be placed on tops of the rows. Interaction of the stress fields initially arranges the "rectangles" within the rows (Figure 12a); then it arranges adjacent rows with respect to one an-other (Figure 12b). The resultant ordered structure is shown in Figure 12c. The described behavior of "rectangles" can be derived from the STM images presented in the previous sections. In addition, investigation of appearance of the RHEED patterns allowed us to conclude that the process of dimer ordering in the *c*(8 × 8) structure is gradual: the pattern reflexes appearing on transition from (2 × 1) to (4 × 4) reach maximum brightness gradually; it means that the *c*(8 × 8) structure does not arise instantly throughout the sample surface, but originally form some nuclei ("standalone rectangles" like those in Figure 5a) on which mobile ad-dimers crystallize in the ordered surface configuration.

### Stability

A source of stability of the Si(001) surface configuration composed by ad-dimers gathered in the rectangular islands has not been found to date. Some of possible sources of stabilization of structures with high-order periodicity were considered in refs. [31,47,51-53]. One of the likely reasons of high-order structure formation might be the non-uniformity of the stress field distribution on a sample surface and dependence of this distribution on such factors as process temperature, sample cooling rate, specimen geometry, and a way of sample fastening to a holder, the presence of impurity atoms on and under the surface. Thus, it is clear only that ad-dimers form "rectangles" which are energetically favorable at temperature conditions of the experiments.

In this connection, a guide for further consideration could be found in ref. [43] where an issue of the critical epinucleus--or the smallest island which unreconstructs the surface and whose probability of growth is greater than the likelihood of decay--on the (2 × 1) reconstructed Si(001) was theoretically investigated. First-principle calculations showed that dimer pairs in epiorientation are metastable and the epinucleus consists of triple dimers [43]. Unfortunately, we failed to observe triple-dimer islands in our experiments, and calculations were limited to three dimers in the cited article. Some formations smaller than "rectangles" sometimes are observed in images of the rarified structures (Figure 5a), but they are likely single dimers (Figure 5b) and dimer pairs. We believe that the short "rectangles that we deal with in this article might be considered as epinuclei for the *c*(8 × *n*) structure because, although they show no tendency to grow by themselves, they are both seeds and structural units for formation of larger islands, such as chains (Figure 5a), grouped chains (Figure 2a), and complete ares (Figure 6). On the other hand, they also do not tend to decay or annihilate even on as powerful sinks as steps (Figure 5a). Thus, we conclude that the stability of such epi-islands as dimer pair-vacancy-pair (short "rectangles", Figure 9a, c) is the highest. Less probable (stable) configuration is pair-vacancy-dimer-vacancy-pair (long "rectangle", Figure 9b). We think its lower stability is due to the presence of a single epi-oriented dimer in the center. That is why long "rectangles" are much less spread on the Si(001) surface than the short ones; and the entire structure stabilization in the presence of the long "rectangles" requires appearance of additional dimer vacancies between "rectangles" in adjacent rows in the vicinity of the long blocks.

### Remark on connection with Ge epitaxial growth

We wish to observe that the temperature interval from 550 to 600°C, in which the reported phase transition occurs, is commonly adopted as a frontier between the so-called low-temperature and high-temperature modes of Ge quantum dot array growth on the Si(001) surface [54]. This means that the low-temperature arrays obtained by MBE usually grow on the *c*(8 ×* n*)-reconstructed Si surface densely covered by the above described "rectangles" if no special precautions are taken to ensure the slow cooling of a Si substrate after surface preparation for Ge deposition. High-temperature arrays always form on the (2 × 1) reconstructed surface. The difference in the initial surface morphology may cause a difference in stress distribution in Ge wetting layer which, in turn, may affect the cluster nucleation and growth. Of course, this hypothesis requires an accurate experimental verification.

## Conclusion

In summary, it may be concluded that the Si(001) surface prepared under the conditions of the UHV MBE chamber in a standard wafer preparation cycle has *c*(8 × *n*) reconstruction which is partly ordered only in one direction. Two types of unit blocks form the rows running along [110] and [] axes. When the long block disturbs the order in a row, a dimmer-vacancy defect appears in the adjacent row in the vicinity of the long block to restore the checkerboard order of blocks in the neighboring rows.

Discrepancies of RHEED patterns and STM images were detected. According to RHEED data, (2 × 1) and (4 × 4) structures can form the Si(001) surface during sample treatment. STM studies of the same samples at room temperature show that a high-order *c*(8 × 8) reconstruction exists on the Si(001) surface; simultaneously, the underlying layer is (2×1) reconstructed in the areas free of the *c*(8 × 8) structure. A fraction of the surface area covered by the *c*(8 × 8) structure decreases as the sample cooling rate is reduced. RHEED patterns corresponding to the (4 × 4) reconstruction arise at ~600°C in the process of sample cooling after annealing. The reconstruction is reversible: the (4 × 4) structure turns into the (2 × 1) one at ~600°C in the process of the repeated sample heating, the (4 × 4) structure appears on the surface again at the same temperature during recurring cooling.

A model of the *c*(8 × 8) structure based on epioriented ad-dimer complexes has been presented. Ordering of the ad-dimer complexes likely arises because of interaction of the stress fields produced by them. The discrepancies of the STM and RHEED data have been explained within the proposed model: the *c*(8 × 8) structure revealed by STM has been evidenced to manifest itself as the (4 × 4) one in the RHEED patterns.

Probable causes for the *c*(8 × 8)-reconstructed Si(001) surface formation have been discussed. A combination of a low temperature of sample annealing and a high rate of its cooling may be considered as one of the most plausible factors responsible for its appearance. The structural units of the studied reconstruction are supposed to be its critical epinuclei.

## Abbreviations

MBE: molecular beam epitaxy; RHEED: reflected high energy electron diffraction; STM: scanning tunneling microscopy; UHV: ultra-high vacuum.

## Competing interests

The authors declare that they have no competing interests.

## Authors' contributions

LA participated in the design of the study, carried out the sample treatments, STM and RHEED measurements, and performed the data analysis; she proposed the structural models; she also supervised the research project (Π2367). VY conceived of the study, participated in its design and coordination, participated in data analysis, discussions and interpretation of the results; he also coordinated the research projects. KC participated in sample treatments and discussions of the results, VS took part in STM experiments and STM data interpretation, MS carried out preliminary and final treatments of the samples, and took part in the discussions. LK carried out chemical treatments of the samples, took part in the discussions. LA and VY wrote the article. All authors read and approved the final manuscript.

## Endnotes

1. The samples were heated over 920°C about a half of the final annealing time; a diagram of the thermal processing and some additional details can be found in ref. [27].

## References

[B1] Yuryev VA Arapkina LV Chapnin VA Kalinushkin VP Kiryanova NV Uvarov OV Chizh KV Krylova LA Stepanov RO Zaytsev OO Development of physical and technological basis of the controllable formation of densely packed Ge nanocluster arrays on the silicon (100) surface by means of ultrahigh vacuum molecular beam epitaxy Report on Research Project 2007-3-1.3-25-01-303 of the Science and Innovations Agency of the Russian Federation, A. M. Prokhorov General Physics Institute of the Russian Academy of Sciences, Moscow, Russia 2007. State Reg. No. 0220.0 802501 (VNITC)

[B2] Hamers RJ Tromp RM Demuth JE Scanning tunneling microscopy of Si(001) Phys Rev B 1986 34 5343 10.1103/PhysRevB.34.5343 9940366

[B3] Chadi DJ Stabilities of single-layer and bilayer steps on Si(001) surfaces Phys Rev Lett 1987 59 1691 10.1103/PhysRevLett.59.1691 10035304

[B4] Hu X Lin Z Hydrogen adsorption induced phase transitions on Si(100)-c(8 × 8): temperature dependence studied by LEED Appl Surf Sci 1995 90 111 10.1016/0169-4332(95)00078-X

[B5] Murray PW Lindsay R Leibsle FM Wincott PL Thornton G Direct observation of the c(8 × 8) defect structure on Si(001) using scanning tunnelling microscopy Phys Rev B 1996 54 13468 10.1103/PhysRevB.54.13468 9985249

[B6] Kubo T Agura T Takagi N Nishijima M Investigation on the surface electronic states of the Si(001) c(4 × 2) and c(8 × 8) surfaces: An electron energy loss spectroscopy study Jpn J Appl Phys 1997 36 L975 10.1143/JJAP.36.L975

[B7] Iton H Ann T Kawasaki T Ichinokawa T Surface structures and growth mode of Cu/Si(100) surfaces by scanning tunnelling microscopy Surf Rev Lett 1998 5 747 10.1142/S0218625X98001122

[B8] Koo JY Yi JY Hwang C Kim DH Lee G Lee S Basic structure of the kinked monatomic steps on the Si(001) surface Phys Rev B 1998 57 8782 10.1103/PhysRevB.57.8782

[B9] Hata K Yasuda S Shigekawa H Reinterpretation of the scanning tunnelling microscopy images of Si(100)-(2 × 1) dimers Phys Rev B 1999 60 8164 10.1103/PhysRevB.60.8164

[B10] Liu BZ Katkov MV Nogami J An STM study of Cu on Si(001) in the c(8 × 8) structure Surf Sci 2000 453 137 10.1016/S0039-6028(00)00329-0

[B11] Okada H Fujimoto Y Endo K Hirose K Mori Y Detailed analysis of scanning tunneling microscopy images of the Si(001) reconstructed surface with buckled dimers Phys Rev B 2001 63 195324 10.1103/PhysRevB.63.195324

[B12] Goryachko A Melnik PV Nakhodkin NG Afanasjeva TV Koval IF New features of the Si(100)-c(4 × 4) reconstruction observed with STM: suggestion of the structure with lowered symmetry Surf Sci 2002 497 47 10.1016/S0039-6028(01)01623-5

[B13] Liu HW Yang HQ Guo HM Wang YL Lin X Pang SJ Gao HJ Patterns formed on the dimer vacancy array of Si(100) by self-assembly Nanotechnol 2002 13 729 10.1088/0957-4484/13/6/306

[B14] Arapkina LV Shevlyuga VM Yuryev VA Structure and peculiarities of the (8 × n)-type Si(001) surface prepared in a molecular beam epitaxy chamber: A scanning tunneling microscopy study JETP Lett 2008 87 215 [ArXiv:0908.1346] 10.1134/S0021364008040085

[B15] Arapkina LV Chizh KV Shevlyuga VM Yuryev VA Aseev AL, Dvurechenskiĭ AV The controllable formation of densely packed arrays of Ge nanoclusters on the silicon (001) surface by means of ultrahigh vacuum molecular beam epitaxy Russian Conference on Actual Problems of Semiconductor Photoelectronics ("Photonics-2008") 2008 Novosibirsk, Russia: A. V. Rzhanov Institute of Semiconductor Physics of Siberian Brunch of the Russian Academy of Sciences 48

[B16] Arapkina LV Yuryev VA Shevlyuga VM STM and RHEED investigations of the c(8 × n) defect structure on Si(001) 25-th International Conference on Defects in Semiconductors (ICDS-25) 2009 St. Petersburg, Russia 348

[B17] Arapkina LV Yuryev VA Chizh KV Chapnin VA Nucleation and growth of Ge hut clusters on the Si(001) surface at low temperatures Proc XIV Int Symp "Nanophysics and nanoelectronics" 2010 2 531 Nizhni Novgorod, Russia: Inst. Microstruct. Phys. RAS

[B18] Dvurechenskii AV Zinoviev VA Kudryavtsev VA Smagina JV Effects of low-energy ion irradiation on Ge/Si heteroepitaxy from molecular beam JETP Lett 2000 72 3 131 10.1134/1.1316815

[B19] Dvurechenskii AV Zinoviev VA Smagina JV Self-organization of an ensemble of Ge nanoclusters upon pulsed irradiation with low-energy ions during heteroepitaxy on Si JETP Lett 2001 74 267 10.1134/1.1417163

[B20] Dvurechenskii AV Zinovyev VA Kudryavtsev VA Smagina JV Novikov PL Teys SA Ion-beam assisted surface islanding during Ge MBE on Si Phys Low-Dim Struct 2002 1/2 303

[B21] Dvurechenskii AV Smagina JV Zinoviev VA Armbrister VA Volodin VA Efremov MD Elemental composition of nanoclusters formed by pulsed irradiation with low-energy ions during Ge/Si epitaxy JETP Lett 2004 79 7 333 10.1134/1.1765177

[B22] Dvurechenskii AV Smagina JV Zinovyev VA Teys SA Gutakovskii AK Modification of growth mode of Ge on Si by pulsed low-energy ion-beam irradiation Int J Nanoscience 2004 3 1/2 19 10.1142/S0219581X04001778

[B23] Dvurechenskii AV Smagina JV Groetzschel R Zinoviev VA Armbrister VA Novikov PL Teys SA Gutakovskii AK Ge/Si quantum dot nanostructures grown with low-energy ion beam-epitaxy Surf Coat Technol 2005 196 1-3 25 10.1016/j.surfcoat.2004.08.082

[B24] Dvurechenskii AV Smagina JV Armbrister VA Zinovyev VA Novikov PL Teys SA Groetzschel R Joyce B, Kelires P, Naumovets A, Vvedensky D Ge/Si nanostructures with quantum dots grown by ion-beam-assisted heteroepitaxy Quantum Dots: Fundamentals, Applications and Frontiers 2005 Netherlands: Kluwer Academic Publishers 135 full_text

[B25] Stepina NP Dvurechenskii AV Armbrister VA Smagina JV Volodin VA Nenashev AV Leitão JP do Carmo MC Sobolev NA MBE growth of Ge/Si quantum dots upon low-energy pulsed ion irradiation Thin Solid Films 2008 517 309 10.1016/j.tsf.2008.08.166

[B26] Smagina JV Zinovyev VA Nenashev AV Dvurechenskiĭ AV Armbrister VA Teys SA Self-assembly of germanium islands under pulsed irradiation by a low-energy ion beam during heteroepitaxy of Ge/Si(100) structures JETP 2008 106 517 10.1134/S1063776108030114

[B27] Arapkina LV Yuryev VA Classification of Ge hut clusters in arrays formed by molecular beam epitaxy at low temperatures on the Si(001) surface Phys Usp 2010 53 3 279 [ArXiv:0907.4770]. 10.3367/UFNe.0180.201003e.0289

[B28] Arapkina LV Yuryev VA Atomic structure of Ge quantum dots on the Si(001) surface JETP Lett 2010 91 6 281 [ArXiv:0908.0883]. 10.1134/S0021364010060056 PMC3211434 21711886

[B29] Arapkina LV Yuryev VA Nucleation of Ge quantum dots on the Si(001) surface Phys Rev B 2010 82 045315 [ArXiv:0907.4665] 10.1103/PhysRevB.82.045315

[B30] Yuryev VA Arapkina LV Defects of Ge quantum dot arrays on the Si(001) surface Physica B 2009 404 4719 [ArXiv:0908.0841] 10.1016/j.physb.2009.08.118

[B31] Miki K Sakamoto K Sakamoto T Is the c(4 × 4) reconstruction of Si(001) associated with the presence of carbon? Appl Phys Lett 1997 71 3266 10.1063/1.120308

[B32] Uhrberg RIG Northrup JE Biegelsen DK Bringans RD Swartz LE Atomic structure of the metastable c(4 × 4) reconstruction of Si(100) Phys Rev B 1992 46 10251 10.1103/PhysRevB.46.10251 10002868

[B33] Zhang Z Kulakov MA Bullemer B Scanning tunnelling microscopy study of Si(100)-c(4 × 4) structure formation by annealing of Si epitaxial films Surf Sci 1996 369 69 10.1016/S0039-6028(96)00925-9

[B34] Zhang Z Kulakov MA Bullemer B Surface morphology and reconstructions of ultra thin Si films growth by solid-phase epitaxy Thin Solid Films 1997 294 88 10.1016/S0040-6090(96)09253-X

[B35] Men FK Erskine JL Metastable oxygen-induced ordered structure on the Si(001) surface Phys Rev B 1994 50 11200 10.1103/PhysRevB.50.11200 9975237

[B36] Eltsov KN Ultrahigh vacuum scanning tunneling microscope STM GPI-300 http://surface.gpi.ru/papers/gpi300e.pdf

[B37] Eltsov KN Klimov AN Kosyakov AN Obyedkov OV Shevlyuga VM Yurov VY Konov VI, Eltsov KN Ultrahigh vacuum scanning funnelling microscope GPI-300 Chemical state and atomic structure of fcc metal surfaces in chemical reaction with halogens, Proc General Phys Inst 2003 59 Moscow, Russia: Nauka 45

[B38] Yurov VY Klimov AN Scanning tunneling microscope calibration and reconstruction of real image--drift and slope elimination Rev Sci Instrum 1994 65 1551 10.1063/1.1144890

[B39] Kiryushina IV Processes of liquid chemical preparation of silicon wafers in the VLSI production with sub-micrometer design rules PhD thesis 2003 JSC Mikron, Zelenograd, Moscow, Russia [In Russian]

[B40] Eltsov KN Shevlyuga VM Yurov VY Kvit AV Kogan MS Sharp tungsten tips prepared for STM study of deep nanostructures in UHV Phys Low-Dim Struct 1996 9/10 7

[B41] Horcas I Fernandez R Gomez-Rodriguez JM Colchero J Gomez-Herrero J Baro AM WSxM: A software for scanning probe microscopy and a tool for nanotechnology Rev Sci Instrum 2007 78 013705 10.1063/1.2432410 17503926

[B42] Mahan JE Geib KM Robinson GY Long RG A review of the geometrical fundamentals of reflection high-energy electron diffraction with application to silicon surfaces J Vac Sci Technol A 1990 8 3692 10.1116/1.576481

[B43] Pala RGS Liu F Critical epinucleation on reconstructed surfaces and first-principle calculation of homonucleation on Si(001) Phys Rev Lett 2005 95 136106 10.1103/PhysRevLett.95.136106 16197156

[B44] Zhang Z Wu F Zandvliet HJW Poelsema B Metiu H Lagally MG Energetics and dynamics of Si ad-dimers on Si(001) Phys Rev Lett 1995 74 3644 10.1103/PhysRevLett.74.3644 10058257

[B45] Brocks G Kelly PJ Car R The energetics of adatoms on the Si(100) surface Surf Sci 1992 269/270 8601 10.1016/0039-6028(92)91362-F

[B46] Ney A Schulz JJ Pampuch C Peripelittchenko L Koch R Atomic structure of low temperature prepared Si(001) substrates Surf Sci 2002 520 L633 10.1016/S0039-6028(02)02296-3

[B47] Aruga T Murata Y Ordered-defect model for Si(001)-(2 × 8) Phys Rev B 1986 34 5654 10.1103/PhysRevB.34.5654 9940400

[B48] Zhang Z Metiu H The self-organization of Si atoms adsorbed on a Si(100) surface: an atomic level kinetic model Surf Sci Lett 1993 292 L781 10.1016/0039-6028(93)90378-W

[B49] Terakura K Yamasaki T Uba T Stich I Atomic and molecular processes on Si(001) and Si(111) surfaces Surf Sci 1997 386 207 10.1016/S0039-6028(97)00315-4

[B50] Mo YW Swartzentruber BS Kariotis R Webb MB Lagally MG Growth and equilibrium structures in the epitaxy of Si on Si(001) Phys Rev Lett 1989 63 2393 10.1103/PhysRevLett.63.2393 10040877

[B51] Yang HQ Zhu CX Gao JN Xue ZQ Pang SJ Large area dimer vacancy array on the Si(100) surface studied by scanning tunneling microscope Surf Sci 1998 412/413 236 10.1016/S0039-6028(98)00431-2

[B52] Natori A Nishiyama R Yasunaga H Stability of ordered missing-dimer structures and the ordering dynamics on Si(001) Surf Sci 1998 397 71 10.1016/S0039-6028(97)00720-6

[B53] Zhao YF Yang HQ Pang SJ Si(100)-c(4 × 8) reconstruction formed in a highly nonequilibrium process Phys Rev B 2000 62 R7715 10.1103/PhysRevB.62.R7715

[B54] Dvurechenskii AV Yakimov AI Quantum dot Ge/Si heterostructures Phys Usp 2001 44 12 1304 10.1070/PU2001v044n12ABEH001057

